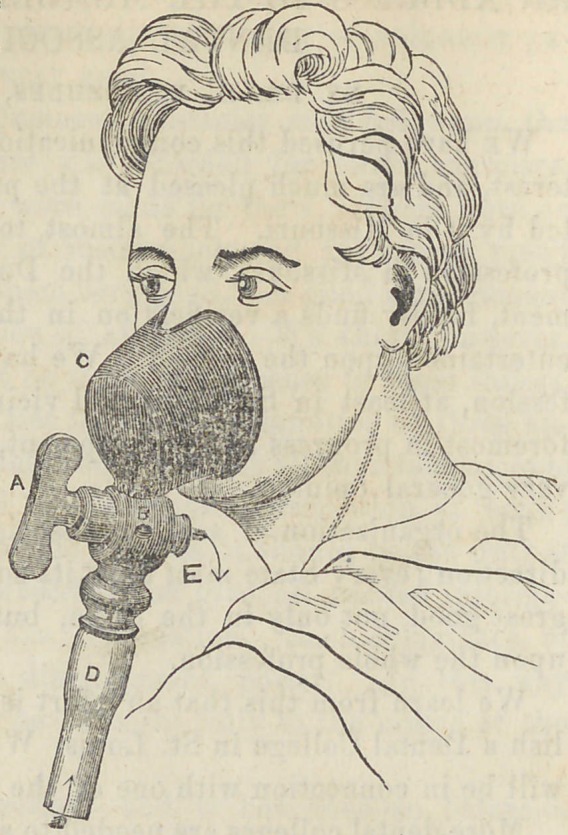# A New Inhaler for All Anæsthetics

**Published:** 1866-09

**Authors:** 


### A NEW INHALER FOR ALL ANÆSTHETICS.

The accompanying cut represents a new inhaler for all anæsthetics, invented by Dr. D. H. Goodwillie, of New York, and, we presume, a very valuable instrument. In addition to the cut, the following description will give a very accurate idea of the instrument:

A, Faucet containing the valves, and revolving quarter of a circle.

B, Face piece, two sizes.

D, Inhalation valve.

E, Exhalation valve.

The superiority of the Inhaler consists in its simplicity, safety, efficiency and economy.

The two face pieces of different sizes will fit all cases. The patient can breathe by the nose or mouth. The mouth can be held open by the face piece if desired.

Direct the patient to open the mouth before the Inhaler is applied to the face, and by a slight pressure the mouth can be kept open during inhalation. The breath (carbonic acid) is thrown off and not rebreathed, thus avoiding asphyxia.

The Inhaler is well adapted to the administration of nitrous oxyde. It is also equally well adapted to the administration of chloroform or ether, as fresh air can be admitted (at B, by revolving faucet A,) in any desired quantity, so essential in giving these anæsthetic agents. Chloroform or ether are inhaled directly from the bottle, by an inhalation cork; none of it is wasted—a great saving compared with the ordinary way of giving it.

## Figures and Tables

**Figure f1:**